# New Medical School

**Published:** 1846-11

**Authors:** 


### NEW MEDICAL SCHOOL.

Among the acts passed by the last legislature of the State of New York, was one incorporating the University of Buffalo. The trustees have organized the Medical Department of the University by establishing seven professorships, and the election of the following gentlemen professors: James Hadley, M. D., Professor of Chemistry and Pharmacy; Charles B. Coventry, M. D., of Physiology and Medical Jurisprudence; James Webster, M. D., of General and Special Anatomy; Charles A. Lee, M. D., of Pathology and Materia Medica; James P. White, M. D., of Obstetrics and Diseases of Women and Children; Frank H. Hamilton, M. D., of Principles of Surgery and Clinical Surgery; Austin Flint, M. D., of Principles and Practice of Medicine and Clinical Medicine.

This list embraces the entire Medical Faculty of Geneva College, with the exception of Dr. Thomas Spencer. We learn that the professors have accepted their appointments with the understanding, that the course of lectures shall be delivered after the course at Geneva has closed, which is about the first of February. The school at Geneva remains as it was; the lectures in it will go on as they have done heretofore, and the course at Buffalo will follow in a different period of the year.

